# Evaluation of the In Vivo Antioxidant Capacity of Sheep Hemoglobin Hydrolysate in a Rat Model

**DOI:** 10.3390/biology15151227

**Published:** 2026-07-23

**Authors:** Yina Qiao, Zhenru Wang, Yilin Yang, Yang Zi, Mingyue Li, Xin Hou, Yingchun Liu, Yujie Guo, Feng Gao

**Affiliations:** 1 College of Animal Science, Inner Mongolia Agricultural University, Hohhot 010018, China; qyn7788@emails.imau.edu.cn (Y.Q.); 13804771148@163.com (Z.W.); y905963976@163.com (Y.Y.); ziyang88992022@126.com (Y.Z.); lmy25256@163.com (M.L.); 16606347227@163.com (X.H.); 2 Inner Mongolia Key Laboratory of Biomanufacturing, College of Life Science, Inner Mongolia Agricultural University, Hohhot 010011, China; yingchun.liu@imau.edu.cn; 3 Key Laboratory of Agro-Products Processing, Ministry of Agriculture and Rural Affairs, Institute of Food Science and Technology, Chinese Academy of Agricultural Sciences, Beijing 100193, China; guoyujie@caas.cn

**Keywords:** sheep hemoglobin, enzymatic hydrolysate, rat, antioxidant function, Nrf2-ARE, NF-κB

## Abstract

Sheep hemoglobin hydrolysate (SHH) has important biological antioxidant functions. However, the specific mechanism is not well understood. The aim of this study was to investigate the antioxidant activity of SHH in vivo and its potential mechanism. Eighteen Sprague Dawley rats were randomly divided into DG (gavaged with 0.9% saline), VC (gavaged with vitamin C 100 mg/kg BW) and SHH (gavaged with SHH 800 mg/kg BW) groups. The rats were given intragastric administration for 21 days. On the 22nd day, all rats were intraperitoneally injected with diquat as a challenge. The results showed that SHH treatment had no obvious effect on the growth performance and main organ index of rats after the diquat challenge. Compared to the DG group, the SHH group maintained higher serum T-AOC and GSH-Px levels after the diquat challenge. SHH significantly increased GSH-Px activity in the liver and kidney, and decreased MDA content in the kidney after the diquat challenge. SHH significantly up-regulated the gene expression of *TrxR* in the liver and *Nrf2* in the kidney but had no significant effect on the expression of *NF-κB* in the kidney. Histopathological observations showed that SHH could improve the morphology of liver cells, and no obvious pathological damage was found in all organs. In conclusion, the relative decrease in oxidative stress markers observed with prophylactic SHH supplementation after diquat challenge may be mediated by the regulation of transcriptional expression of Nrf2-related redox genes. These findings demonstrate SHH’s potential as a natural antioxidant and provide promising strategies for converting low-value meat industry byproducts into functional supplements.

## Simple Summary

Oxidative stress impairs animal health and lowers productivity. Bioactive peptides from animal by-products represent a potential natural approach to reducing oxidative damage. In this study, we tested the in vivo antioxidant capacity of an enzymatically produced sheep hemoglobin hydrolysate (SHH) in a diquat-challenged rat model. It raised the total antioxidant capacity and boosted key antioxidant enzyme activities in the blood, liver, and kidneys. SHH also lowered lipid peroxidation, especially in the kidneys, and may trigger protective Nrf2 antioxidant-related genes. These results suggest that sheep hemoglobin hydrolysate possesses promising antioxidant potential under acute stress conditions and could be developed as a functional feed ingredient to support animal health.

## 1. Introduction

Oxidative stress is a pathological state characterized by an imbalance between antioxidants and oxidants, which disrupts redox signaling and causes molecular damage [1,2]. Nutritional deficits, environmental challenges, and physiological stressors can induce metabolic syndrome and oxidative stress, which promotes the excessive production of reactive oxygen species (ROS) [3,4]. When an animal experiences mild oxidative stress, its inherent antioxidant defense system acts as a survival mechanism to eliminate free radicals and protect against external damage [5,6]. However, persistent or severe stress can overwhelm cellular defenses, leading to sustained elevations in ROS and consequent oxidative stress [7]. Recent studies have established a link between oxidative stress and diverse pathologies, including liver [8] and kidney injury [9], immune dysfunction [10], and intestinal epithelial barrier disruption [11]. These impairments compromise animal health and physiological resilience, particularly under nutritional or environmental challenges, by increasing susceptibility to diseases and reducing overall vitality. Diquat is a widely used oxidative stress inducer that causes significant damage to the liver, kidneys, and intestines, making it a standard tool for investigating antioxidant mechanisms.

Recently, bioactive peptides derived from animals have attracted much attention due to their antibacterial, antihypertensive, anti-inflammatory, immunomodulatory and other physiological activities [12]. These peptides, which are typically low-molecular-weight peptides (length between 2~30 amino acids) with molecular weights typically under 6 kDa [13,14], exhibit specific biological functions, among which antioxidant peptides are the most extensively studied [15,16]. These peptide-based substances exert antioxidant effects through multiple mechanisms, including free radical scavenging, the upregulation of antioxidant enzymes, and the modulation of signaling pathways such as the nuclear factor erythroid 2-related factor 2 (*Nrf2*) and endogenous antioxidant defense and nuclear factor kappa-B (*NF-κB*) pathways [17]. Once these pathways are activated, *Nrf2* drives the expression of downstream cytoprotective enzymes such as superoxide dismutase (SOD) and glutathione peroxidase (GPX) [18]. As a classical biomarker for assessing cellular redox homeostasis, SOD and GPX neutralizes toxic superoxide radicals and hydrogen peroxide, respectively, to prevent lipid peroxidation [19,20]. In addition, heme oxygenase-1 (*HO-1*) degrades the prooxidant free heme to antioxidant metabolites, while thioredoxin reductase (*TrxR*) maintains redox balance by regenerating the thioredoxin system and jointly relieves oxidative stress [21]. Blood-derived proteins like hemoglobin represent a rich natural source of precursors for such antioxidant peptides [22]. Enzymatic hydrolysis of these proteins releases numerous short peptides, which have both antioxidant and anti-inflammatory activities [23]. Sheep blood is a high-volume byproduct of the slaughter industry but remains underutilized, typically treated as a low-value material due to its high pollutant load and disposal costs [24]. However, utilizing sheep blood as a high-quality protein resource or as a feed additive for animal nutrition and feed is one way to increase its value. The development of high-value functional peptides from this source therefore presents considerable industrial potential. Although hydrolysates of porcine and bovine blood proteins have been studied and proven to possess strong antioxidant properties [23,25], the antioxidant effects and underlying mechanisms of sheep hemoglobin hydrolysate (SHH) in vivo remain largely unexplored. Specifically, it is currently unknown how dietary SHH modulates endogenous antioxidant networks under acute oxidative stress in vivo. We hypothesized that prophylactic dietary supplementation with SHH would enhance the antioxidant capacity in rats and potentially regulate the activation of related antioxidant reactions. Consequently, this study aimed to evaluate the antioxidant protective effect of SHH after a diquat challenge by comparing antioxidant indices in the serum and major organs. And, we explore whether SHH functions through the endogenous antioxidant system and related signaling pathways, which provides a scientific basis for the use of SHH as a green feed additive and functional supplement.

## 2. Materials and Methods

### 2.1. Animals and Treatments

All experimental protocols were approved by the Animal Care and Ethics Committee of Inner Mongolia Agricultural University (approval number: NND20241191). The procedures strictly adhered to the institutional guidelines for the care and use of laboratory animals in China. Sheep hemoglobin hydrolysate was prepared in the laboratory via enzymatic hydrolysis.

Lyophilized sheep hemoglobin powder was dissolved in distilled water at a substrate concentration of 5%. Commercial protease (animal proteolytic enzyme, Nanning Pangbo Biotechnology Co., Ltd., Hong Kong, China) was then added at an amount of 3% of the matrix mass. The obtained mixture was prepared in a shaking incubator at 50 °C and pH 6.5 for 3 h. The SHH degree of hydrolysis was 39.09% and the yield of peptides was 55.82%. After hydrolysis, the hydrolysate was boiled at 100 °C for 10 min to terminate the enzymatic reaction. The hydrolysate was freeze-dried and stored at−40 °C until needed.

Eighteen healthy SD rats, aged 7–8 weeks old and weighing 250 ± 5 g on average, were purchased from the Animal Center of Inner Mongolia University (Hohhot, China). The rats consisted of 9 males and 9 females that were equally distributed across the three groups. All rats’ housing, cages, water bottles, and related equipment were thoroughly disinfected before use. The experimental diet was sourced from Inner Mongolia Bole Biological Co., Ltd. (Chifeng, China), and its nutritional composition is detailed in Table 1. The animals had ad libitum access to food and water, and their bedding was changed regularly. Ventilation was also provided. After a seven-day acclimatization period, the rats were randomly assigned to three groups with six replicates based on average body weight: the diquat-model group (diquat challenged group, DG) received 0.9% saline; the positive group (VC) was gavaged with 100 mg of vitamin C per kg BW and served as the standard control [26]; and the sheep hemoglobin hydrolysate group (SHH) was gavaged with 800 mg of sheep hemoglobin hydrolysate per kg BW [27]. On the final day, all rats were intraperitoneally injected with 0.1 mmol of diquat per kg BW as a challenge [26,28]. During the treatment period, body weight was recorded every five days, and the total rearing period was 21 days. The rats were slaughtered 24 h after challenge administration and blood samples were collected.

### 2.2. Sample Collection

Twenty-four hours after the diquat challenge, a blood sample was gently collected from the tail vein and placed into a sterile coagulation-promoting tube. The rats were fasted for 12 h before blood sampling. All collected blood samples were allowed to stand at room temperature for 30 min to allow for complete coagulation. Subsequently, the samples were centrifuged at 3000× *g* at 4 °C for 15 min in a refrigerated centrifuge. Separated serum was stored immediately at −80 °C and used in the subsequent analysis. The liver, kidney, spleen, and ileum of rats were dissected after anesthesia with pentobarbital sodium. The remaining tissues were flash-frozen in liquid nitrogen and stored at −80 °C. A portion of each tissue was used to prepare a 10% (*w*/*w*) homogenate. This homogenate was centrifuged at 3500× *g* for 10 min. The resulting supernatant was collected for subsequent quantification of relevant indicators using commercial antioxidant assay kits.

### 2.3. Histological Analysis

Liver, kidney, spleen, and ileum samples were fixed in 10% formaldehyde for at least 24 h. The fixed tissues were then embedded in paraffin and sectioned to a thickness of 4~6 µm. These sections were stained with hematoxylin and eosin (HE) using a commercial kit (B1002, Wuhan Technology, Wuhan, China) for histopathological analysis. Finally, the stained sections were examined and imaged with a Leica DM750 microscope (Leica, Heidelberg, Germany).

### 2.4. T-AOC, GSH-Px, T-SOD, and MDA Contents in Serum and Organs of Rats

Serum total antioxidant capacity (T-AOC), and serum levels of glutathione peroxidase (GSH-Px), total superoxide dismutase (T-SOD), and malondialdehyde (MDA) were measured using commercial assay kits (T-AOC: A015-2-1; GSH-Px: A005-1-2; T-SOD: A001-1-2; MDA: A003-1-2) from the Nanjing Institute of Jiancheng Biological Engineering (Nanjing, China), following the manufacturer’s protocols. A 0.5 g sample of liver, kidney, or ileum tissue was precisely weighed and homogenized separately in nine volumes of normal saline to yield a 10% homogenate. After centrifugation, the supernatant was collected and diluted with normal saline to an optimal concentration. The resulting supernatants from the liver, kidney, and ileum homogenates were subsequently assayed for MDA, SOD, T-AOC, and GSH-Px levels. These assays were performed using the aforementioned commercial kits according to the manufacturer’s instructions.

### 2.5. Real-Time Polymerase Chain Reaction Analysis

Total RNA was isolated from the liver, kidney and ileum tissues with the RNAiso Plus reagent (Cat. No. 9108, Takara, Japan) according to the manufacturer’s protocol. Reverse transcription was then carried out using the PrimeScript RT kit (Cat. No. RR037A, Takara, Japan) following the manufacturer’s instructions. Quantitative PCR employed SYBR Premix Ex Taq (Cat. No. RR420A, Takara, Japan) and a LightCycler 480 system (Roche, Switzerland). All specific primer sequences were synthesized by Sangon Biotech (Shanghai, China) and are detailed in Table 2. Gene expression levels were normalized to Actb mRNA and calculated via the 2^−ΔΔCt^ method [29].

### 2.6. Statistical Analysis

The data were organized in Excel (Microsoft Corporation, Redmond, WA, USA) and analyzed with SAS version 9.2 (SAS Institute Inc., Cary, NC, USA) using a one-way ANOVA followed by Tukey’s post hoc test; the body weight data were analyzed using repeated-measures ANOVA. The results are presented as means with their standard errors (SEMs). Statistical significance was defined as *p* < 0.05, while *p* < 0.01 was considered highly significant.

## 3. Results

### 3.1. The Feed Intake and Body Weight of Rats

As shown in Table S1, throughout the experimental period, the groups did not exhibit significant changes in food intake or body weight (*p* > 0.05).

### 3.2. The Organ Weight and Index of Rats

The effects of sheep hemoglobin hydrolysate on organ weight and index in rats are presented in Table S2. There were no significant changes in liver, kidney, spleen, and ileum weight or index (*p* > 0.05).

### 3.3. Histological Assessment of Rat Organs

**Figure 1:**
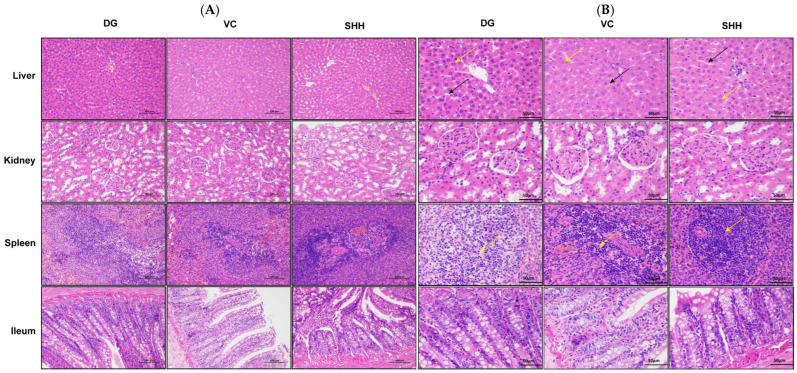
Effects of sheep hemoglobin hydrolysate on organ structure of diquat-challenged rats. The images from left to right are from the DG, VC, and SHH groups. The images from top to bottom show HE staining for structure of the liver, kidneys, spleen, and ileum. (**A**) Magnification: 200×, bars = 100 μm. (**B**) Magnification: 400×, bars = 50 μm. Black arrow: cytoplasm; yellow arrow: nucleus.

The effects of sheep hemoglobin hydrolysate on rat organ structure are shown in Figure 1. In the DG group, the liver tissue displayed loose cytoplasm (black arrow) and contained nuclei that were darker and enlarged (yellow arrow). Hepatocytes in the VC group were neatly arranged, exhibited normal morphology, and possessed prominent nucleoli and abundant cytoplasm. The SHH group showed rounded, plump hepatocytes without obvious abnormalities. In the diquat-challenged group, spleen cell nuclei appeared lightly stained with looser cytoplasm, whereas in the VC and SHH groups, the nucleoli were more darkly stained and the cytoplasm was denser. Furthermore, the kidney and ileum tissues from all three experimental groups exhibited clear staining.

### 3.4. The Antioxidant Capacity of Rat Serum

As shown in Table 3, compared with the diquat-challenged group, serum T-AOC (*p* < 0.01) and GSH-Px levels (*p* < 0.05) were significantly elevated in the VC and SHH groups. Compared to the DG group, the MDA content was markedly lower in the VC group (*p* < 0.05) and no significant differences were observed in the SHH group followed the diquat challenge (*p* > 0.05). There were no significant differences in T-SOD levels among the three groups (*p* > 0.05).

### 3.5. The Antioxidant Capacity of Rat Organs

The VC-treated group showed significantly elevated levels of T-SOD (*p* < 0.05) and GSH-Px (*p* < 0.01) in the liver and the SHH group exhibited a significant increase in GSH-Px content (*p* < 0.05) compared to the positive control group, as shown in Table 4. The T-AOC and MDA levels in the liver did not differ significantly across the three groups (*p* > 0.05). Following the addition of sheep hemoglobin hydrolysate, rat kidney T-SOD (*p* < 0.01) and GSH-Px (*p* < 0.05) levels increased significantly, while MDA levels decreased substantially (*p* < 0.01). T-AOC levels did not show a statistically significant change (*p* > 0.05). Compared to the positive control group, the VC group exhibited significantly elevated T-AOC, T-SOD, and GSH-Px levels (*p* < 0.01), alongside a marked reduction in MDA levels (*p* < 0.01). No significant alterations in ileal T-AOC, GSH-Px, or MDA levels were observed across the groups (*p* > 0.05). The T-SOD level was significantly higher in the VC group than in the other two groups (*p* < 0.01) (Table 4).

### 3.6. mRNA Expression of Genes Involved in Nrf2-ARE and NF-κB Signaling Pathways in Rat Organs

**Figure 2:**
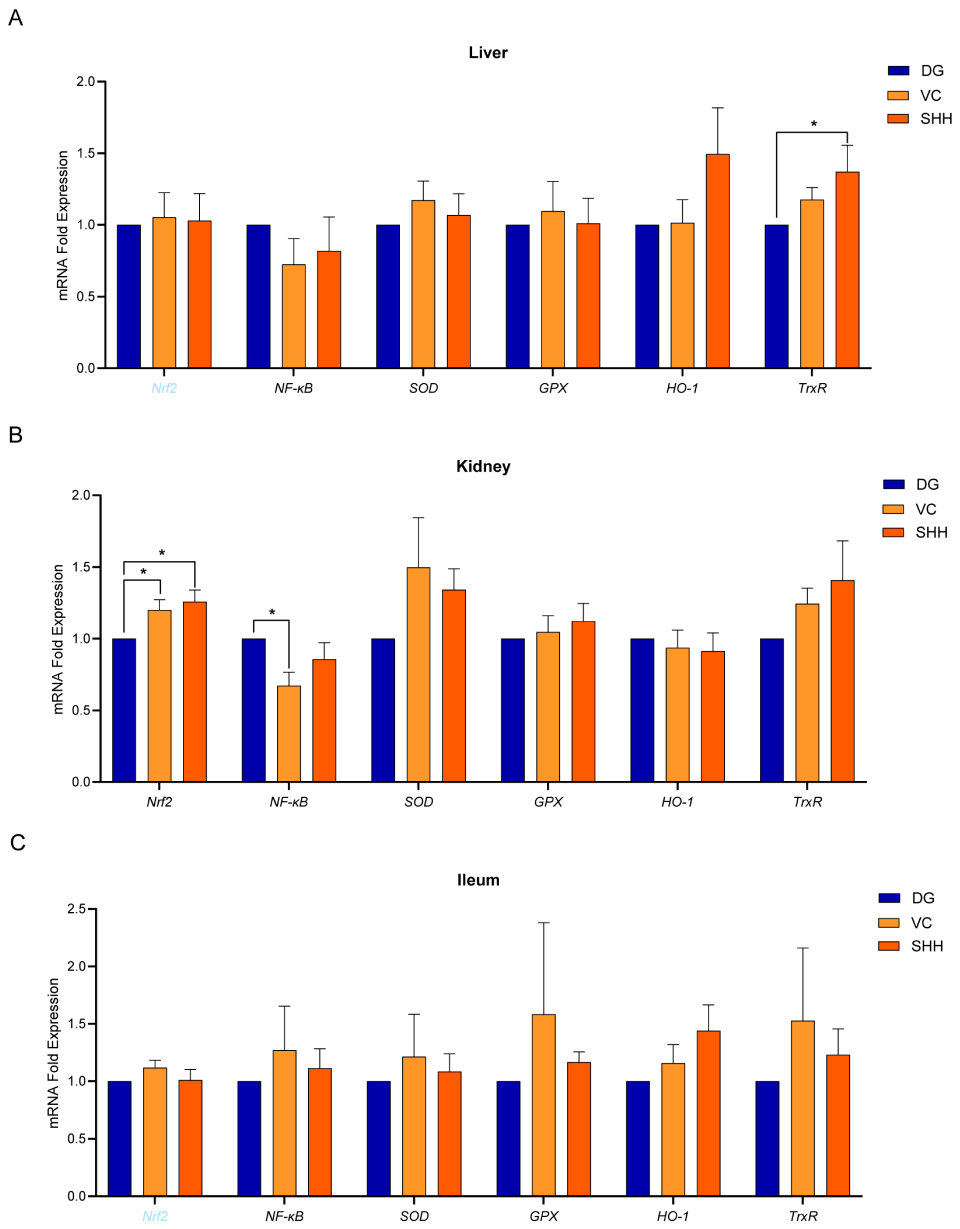
Effects of sheep hemoglobin hydrolysate on mRNA levels of genes involved in *Nrf2-ARE* and *NF-κB* signaling pathways in organs of diquat-challenged rats. (**A**) Effects of SHH on mRNA levels of liver in diquat-challenged rats; (**B**) Effects of SHH on mRNA levels of kidney in diquat-challenged rats; (**C**) Effects of SHH on mRNA levels of ileum in diquat-challenged rats. Gene expression is standardized relative to *ACTB* mRNA levels; *n* = 6. The data are presented as the mean ± SEM. Significant differences between the DG group, VC group, and SHH group: * *p* < 0.05. Abbreviations: *Nrf2*, nuclear factor erythroid 2-related factor 2; *NF-κB,* nuclear factor kappa-B; *SOD*, superoxide dismutase; *GPX*, glutathione peroxidase; *HO-1*, heme oxygenase-1; *TrxR*, thioredoxin reductase.

The effects of sheep hemoglobin hydrolysate on mRNA levels of genes involved in the *Nrf2-ARE* and *NF-κB* signaling pathways in organs of rats are shown in Figure 2. In the liver, *TrxR* gene expression was significantly higher in the SHH group than in the positive control group (*p* < 0.05) (Figure 2A). In the kidney, compared to the positive control group, *NF-κB* gene expression was significantly lower in the VC group (*p* < 0.05). *Nrf2* gene expression in the kidney was significantly elevated compared to the positive control group (*p* < 0.05) (Figure 2B). There were no significant changes in gene expression detected in the ileum across the experimental groups (*p* > 0.05) (Figure 2C).

## 4. Discussion

Animal by-product bioactive peptides have superior biosafety, good gastrointestinal bioavailability and multi-target physiological regulation functions, and are considered to be highly effective natural substitutes for synthetic antioxidants [30,31,32,33]. The antioxidant potential of blood-derived peptides has been widely reported, mainly focusing on hemoglobin hydrolysates from pigs, cattle, and chickens. These studies have demonstrated that blood-derived hydrolysates have strong free radical-scavenging and metal-chelating activities in vitro [25,34,35], and are effective as natural antioxidants in food systems to prevent lipid oxidation in meat products [36,37]. However, most of these studies remain limited to in vitro and cytometry or food preservation applications, with a significant lack of in vivo evaluation in mammalian models. Our studies indicated that sheep hemoglobin hydrolysate administration did not significantly affect feed intake, body weight, or the weight and index of major organs (liver, kidney, spleen, ileum) in rats. This demonstrates the favorable biosafety profile of SHH at a dose of 800 mg/kg BW, which did not induce pathological organ hyperplasia or atrophy. These findings establish a safe foundation for subsequent investigations into its bioactivity. Furthermore, SHH treatment did not produce any discernible histopathological alterations in the major organs, which aligns with prior reports on the safety of blood protein hydrolysates in rodents [38]. Although H&E staining was used to assess overall macroscopic histopathological damage, it cannot directly show the spatial distribution of oxidative stress markers. The lack of histological quantification and specific staining or immunohistochemistry (IHC) in this study limits our understanding of the localization of specific proteins within tissues.

On this basis, we also studied the effect of SHH on serum antioxidation. Our results indicated that serum T-AOC and GSH-Px levels increased significantly following the diquat challenge. This demonstrates that SHH effectively and durably enhanced the antioxidant capacity, demonstrating a particular protective potential against acute oxidative stress. Current mechanistic studies emphasize that this long-lasting in vivo effect is usually driven by specific short-chain peptides [39,40,41], which not only directly quench reactive oxygen species but also act as potent signaling molecules to sustain endogenous antioxidant networks [42,43]. Enzymatic hydrolysis of blood proteins is known to generate numerous low-molecular-weight peptides, which are frequently enriched in hydrophobic, aromatic (such as Tyr, Phe, and Trp), or sulfur/carboxyl-containing amino acids [23,44]. Although no specific peptide sequence of SHH was identified in this study, we hypothesized that the observed antioxidant protection from SHH may be related to bioactive peptides composed of these amino acids. These residues readily react with free radicals by donating hydrogen atoms or electrons, thereby scavenging the radicals [45]. This direct scavenging action consequently reduces the oxidative load. At the same time, antioxidant peptides can enhance the body’s resistance to oxidative challenges by modulating its endogenous antioxidant defense system, primarily through inducing or promoting the expression and activity of enzymes such as SOD and GSH-Px. Research on protein hydrolysates derived from plants, marine organisms, and animal tissues demonstrates that enzymatically liberated peptides can elevate SOD and GSH-Px activities while suppressing MDA production [46,47,48]. Some studies indicate that it is highly possible that hydrolyzed peptides, particularly those potentially containing carboxyl, sulfhydryl, or aromatic residues, can chelate metal ions [46]. This chelation could be critical for inhibiting the formation of metal-catalyzed free radical scavengers and impeding the conversion of hydrogen peroxide to hydroxyl radicals, thereby potentially reducing reactive oxygen species production at its source [49]. Consequently, it diminishes lipid peroxidation, as reflected by the reduced malondialdehyde levels. The GSH-Px content demonstrated that the effect of SHH was slightly weaker than that of VC, and the trend was consistent. These results confirm the nutritional antioxidant potential of SHH. Moreover, the lack of in-depth polypeptidomic characterization (such as LC-MS/MS analysis or precise amino acid composition analysis) limited our ability to correlate the observed antioxidant efficacy with specific active peptide sequences. While assessing complete hydrolysate mixtures is highly relevant for developing cost-effective functional feed additives, any mechanistic associations with specific amino acid motifs remain speculative at this stage. Therefore, isolating the core bioactive peptides and understanding their bioavailability will be our main focus in future research.

We also investigated the organ-specific antioxidant effects of SHH. The results showed that SHH significantly increased liver GSH-Px activity but did not have any significant effects on T-SOD, T-AOC, or MDA levels. This suggests that SHH likely exerts its hepatic antioxidant effect primarily by enhancing the glutathione-based detoxification system, which is consistent with the liver’s role as the principal site of glutathione synthesis and the central organ for maintaining oxidative stress homeostasis [50]. SHH significantly increased T-SOD and GSH-Px while decreasing MDA in the kidney. These results indicate that the kidney is highly responsive to the antioxidant protection conferred by SHH, which effectively alleviated oxidative damage. SHH enhances the renal enzymatic antioxidant system while also reducing free radical- and ROS-induced lipid peroxidation. This dual protective effect is commonly observed in antioxidant function studies of hydrolyzed peptides [51]. However, SHH did not have any significant effects on the antioxidant capacity of the ileum, suggesting that its active components may primarily exert their effects via the circulation and metabolic organs following digestion and absorption [52]. This difference may be the result of a combination of factors. The kidneys are the main sites for the accumulation and excretion of diquat [53]. After absorption, diquat can show high tissue concentrations in the kidneys, making the kidneys particularly sensitive to diquat-related oxidative damage [9]. SHH and its related bioactive peptides are limited in vivo by their short half-lives and local microenvironment-dependent distribution, so their exposure levels in different organs may be uneven. Intestinal epithelium may show relatively stronger tolerance to systemic diquat strikes due to higher renewal rates and barrier protection mechanisms, but this still requires further experimental validation [54]. Future research will incorporate advanced biomarkers such as ROS levels, 8-OHdG, and GSH/GSG ratios, enabling a deeper understanding of the mechanisms of DNA and protein oxidation.

The results showed that SHH treatment significantly upregulated the gene expression level of thioredoxin reductase (TrxR) in the liver. TrxR is a core enzyme within the antioxidant thioredoxin (Trx) system [55]. This system acts as the core of the cell’s antioxidant defense network and is able to remove peroxide and maintain the reduced state of proteins [56]. TrxR is a classical target driven by antioxidant response elements (AREs) [57]. Additionally, Nrf2 regulates the gene expression of enzymes involved in glutathione metabolism as well as antioxidants [58]. The significant increase in GSH-Px activity and the upregulation of TrxR in liver tissue indicate that SHH may activate the Nrf2-ARE signaling pathway in the liver. SHH treatment significantly upregulated Nrf2 expression and may activate the Nrf2 pathway in the kidney. T-SOD and GSH-Px activities were significantly increased while MDA content decreased, collectively indicating an enhanced overall antioxidant capacity. In this experimental model, VC treatment significantly reduced NF-κB gene expression in the kidney, aligning with VC’s established antioxidant activity. There were no significant changes in genes associated with either pathway in the ileum, which was consistent with no statistical changes in T-AOC, GSH-Px and MDA levels. This study initially clarified that the antioxidant effect of SHH may be associated with activation of Nrf2-related antioxidant responses, but the active ingredient and protein mechanism are not clear. In the future, incorporating multiple stable reference genes for enhanced normalization accuracy and integrating IHC or immunofluorescence techniques and Western blotting to precisely localize target protein expression (such as Nrf2) within specific organ structures will be a key focus for our future research. In addition, the isolation and identification of key antioxidant peptides and verification of their signaling pathways at the cellular and animal levels will be key steps in developing them as a novel antioxidant nutritional intervention strategy. A major limitation of the present study is the absence of a healthy control group without a diquat challenge. Consequently, the baseline antioxidant parameters of the rats could not be evaluated, and the absolute severity of the diquat-induced oxidative damage remains unquantified. Future studies must incorporate a normal control group to fully assess the preventive and therapeutic efficacy of SHH. Additionally, the sample size in this study was relatively small. While the current approach complies with the ethical goal of minimizing animal use, it may limit the statistical power to detect subtle changes in highly variable oxidative stress biomarkers. Larger sample sizes and a priori power calculations should be utilized in future validation studies.

## 5. Conclusions

In summary, prophylactic oral administration of SHH showed excellent safety in vivo and promising antioxidant potential by maintaining a higher antioxidant defense capacity compared to the diquat-challenge model groups. This antioxidant response showed an organ-specific pattern, with the kidney showing the most pronounced relative improvement in redox markers. SHH may differentially regulate the expression and activity of downstream antioxidant enzymes through tissue-specific activation of the Nrf2-related antioxidant responses, which is the key to its efficacy.

## Figures and Tables

**Table 1:** Ingredients and nutrient composition of the experimental diets (dry matter basis).

Ingredient	Content (%)
Corn	49.50
Wheat	14.80
Soybean meal	22.10
Imported fish meal	4.10
Imported chicken meal	3.20
Soybean oil	3.00
Premix	1.00
Calcium hydrogen phosphate	2.30
Total	100.00
Nutrient composition	
Dry matter	89.28
Crude protein	23.60
Crude fat	4.31
Crude ash	8.00
Calcium	1.00
Phosphorus	0.60
Crude fiber	5.00
Nitrogen-free extract	48.37

**Table 2:** RT-qPCR primer sequences.

Gene	Sequence (5′-3′)		Length (bp)	GenBank Accession No.
*Nrf2*	F:	GCCTTCCTCTGCTGCCATTAGTC	110	NM_031789.2
	R:	TGCCTTCAGTGTGCTTCTGGTTG		
*NF-κB*	F:	TGTGGTGGAGGACTTGCTGAGG	138	NM_001276711.1
	R:	AGTGCTGCCTTGCTGTTCTTGAG		
*SOD*	F:	GCGGCTTCTGTCGTCTCCTTG	84	NM_017050.1
	R:	GCCTTCATCGCCATGCTTCCC		
*GPX*	F:	TAGGTCCAGACGGTGTTCCAGTG	131	NM_030826.4
	R:	CCAAGCCCAGATACCAGGAATGC		
*HO-1*	F:	CAGACAGAGTTTCTTCGCCAGAGG	129	NM_012580.2
	R:	TGTGAGGACCCATCGCAGGAG		
*TrxR*	F:	TTGCTTGCTTCCTTGTCCACACC	135	NM_001351981.1
	R:	AGCCTTCTTCTACCTGCCACCTC		
*β-actin*	F:	ACTGCCGCATCCTCTTCCTC	86	NM_031144.2
	R:	CATCGGAACCGCTCATTGCC		

**Table 3:** Effects of sheep hemoglobin hydrolysate on antioxidant indices in serum of diquat-challenged rats.

Item ^1^	DG ^2^	VC	SHH	*p*-Value
T-AOC (mmol/L)	0.41 ± 0.03 ^c^	0.58 ± 0.02 ^a^	0.56 ± 0.03 ^a^	0.0007
T-SOD (U/mL)	22.47 ± 0.43	23.58 ± 0.18	22.93 ± 0.44	0.1130
GSH-Px (U/mL)	191.52 ± 11.73 ^b^	233.33 ± 10.69 ^a^	229.41 ± 7.69 ^a^	0.0234
MDA (nmol/mL)	5.68 ± 0.35 ^a^	4.16 ± 0.39 ^b^	5.10 ± 0.32 ^ab^	0.0301

^1^ Data are presented as mean ± standard error. Values in the same column with different lowercase superscript letters (a, b, and c) are significantly different (*p* < 0.05), whereas those with no letter display are not significantly different (*p* > 0.05)., *n* = 6. ^2^ Abbreviations: DG, diquat-challenged group; VC, standard control group; SHH, sheep hemoglobin hydrolysate group; T-AOC, total antioxidant capacity; T-SOD, total superoxide dismutase; GSH-Px, glutathione peroxidase; MDA, malondialdehyde.

**Table 4:** Effects of sheep hemoglobin hydrolysate on organs’ antioxidant indices in diquat-challenged rats.

Item ^1^	DG ^2^	VC	SHH	*p*-Value
Liver				
T-AOC (mmol/g prot)	0.12 ± 0.005	0.11 ± 0.007	0.11 ± 0.007	0.1064
T-SOD (U/mg prot)	5.76 ± 0.27 ^b^	6.94 ± 0.37 ^a^	6.22 ± 0.31 ^ab^	0.0485
GSH-Px (U/mg prot)	98.93 ± 3.30 ^c^	111.14 ± 0.98 ^a^	105.24 ± 0.40 ^ab^	0.0023
MDA (nmol/mg prot)	7.87 ± 1.40	7.19 ± 0.80	7.50 ± 0.89	0.9038
Kidney				
T-AOC (mmol/g prot)	0.02 ± 0.003 ^c^	0.11 ± 0.012 ^a^	0.04 ± 0.002 ^c^	<0.0001
T-SOD (U/mg prot)	5.95 ± 0.23 ^c^	10.62 ± 0.52 ^a^	7.30 ± 0.43 ^b^	<0.0001
GSH-Px (U/mg prot)	113.12 ± 1.79 ^c^	136.55 ± 3.91 ^a^	117.96 ± 2.71 ^b^	0.0001
MDA (nmol/mg prot)	8.81 ± 0.51 ^a^	6.12 ± 0.33 ^c^	6.33 ± 0.43 ^c^	0.0008
Ileum				
T-AOC (mmol/g prot)	0.32 ± 0.01	0.33 ± 0.01	0.29 ± 0.05	0.7609
T-SOD (U/mg prot)	8.20 ± 1.34 ^c^	19.11 ± 2.80 ^a^	12.90 ± 1.22 ^bc^	0.0050
GSH-Px (U/mg prot)	136.47 ± 6.54	168.15 ± 22.81	159.15 ± 19.29	0.4451
MDA (nmol/mg prot)	1.89 ± 0.33	1.14 ± 0.11	1.65 ± 0.55	0.3707

^1^ Data are presented as mean ± standard error. Values in the same column with different lowercase superscript letters (a, b, and c) are significantly different (*p* < 0.05), whereas those with no letter display are not significantly different (*p* > 0.05)., *n* = 6. ^2^ Abbreviations: DG, diquat-challenged group; VC, standard control group; SHH, sheep hemoglobin hydrolysate group; T-AOC, total antioxidant capacity; T-SOD, total superoxide dismutase; GSH-Px, glutathione peroxidase; MDA, malondialdehyde.

## Data Availability

The datasets from this study can be made available upon reasonable request to the corresponding author.

## Supplementary Materials

The following supporting information can be downloaded at https://www.mdpi.com/article/10.3390/biology15151227/s1. Table S1. Effects of sheep hemoglobin hydrolysate on feed intake and body weight in diquat challenged rats; Table S2. Effects of sheep hemoglobin hydrolysate on organs weight and index in diquat challenged rats.

## Abbreviations

The following abbreviations are used in this manuscript:

SD	Sprague Dawley
SHH	Sheep hemoglobin hydrolysate
ROS	Reactive oxygen species
BW	Body weight
HE	Hematoxylin and eosin
T-AOC	Total antioxidant capacity
GSH-Px	Glutathione peroxidase
T-SOD	Total superoxide dismutase
MDA	Malondialdehyde
Nrf2	Nuclear factor erythroid 2-related factor 2
NF-κB	Nuclear factor kappa-B
SOD	Superoxide dismutase
GPX	Glutathione peroxidase
HO-1	Heme oxygenase-1
TrxR	Thioredoxin reductase

## References

[1] Qi H., Wang Y., Fa S., Yuan C., Yang L. (2021). Extracellular Vesicles as Natural Delivery Carriers Regulate Oxidative Stress Under Pathological Conditions. Front. Bioeng. Biotechnol..

[2] Sies H. (2015). Oxidative stress: A concept in redox biology and medicine. Redox Biol..

[3] Heidari H., Lawrence D.A. (2023). Climate Stressors and Physiological Dysregulations: Mechanistic Connections to Pathologies. Int. J. Environ. Res. Public Health.

[4] Petrosyan E., Fares J., Fernandez L.G., Yeeravalli R., Dmello C., Duffy J.T., Zhang P., Lee-Chang C., Miska J., Ahmed A.U. (2023). Endoplasmic Reticulum Stress in the Brain Tumor Immune Microenvironment. Mol. Cancer Res..

[5] Tie H., Xie X., Zhang Z., Zhang J., Xu L., Ruan H., Wu T., Zhang H. (2025). E’jiao and Cubilose Formula Induced Antioxidant Activity and Improved Collagen Expression of Human Skin Fibroblasts During Oxidation Damage. J. Cosmet. Dermatol..

[6] Okagu I.U., Udenigwe C.C. (2022). Transepithelial transport and cellular mechanisms of food-derived antioxidant peptides. Heliyon.

[7] Ferraresso R.L., de Oliveira R., Macedo D.V., Nunes L.A., Brenzikofer R., Damas D., Hohl R. (2012). Interaction between overtraining and the interindividual variability may (not) trigger muscle oxidative stress and cardiomyocyte apoptosis in rats. Oxid. Med. Cell Longev..

[8] Chen J., Su Y., Lin R., Lin F., Shang P., Hussain R., Shi D. (2021). Effects of Acute Diquat Poisoning on Liver Mitochondrial Apoptosis and Autophagy in Ducks. Front. Vet. Sci..

[9] Wu Y., Cui S., Wang W., Jian T., Kan B., Jian X. (2022). Kidney and lung injury in rats following acute diquat exposure. Exp. Ther. Med..

[10] Morris G., Gevezova M., Sarafian V., Maes M. (2022). Redox regulation of the immune response. Cell Mol. Immunol..

[11] Cao S., Wu H., Wang C., Zhang Q., Jiao L., Lin F., Hu C.H. (2018). Diquat-induced oxidative stress increases intestinal permeability, impairs mitochondrial function, and triggers mitophagy in piglets. J. Anim. Sci..

[12] Lu J., Shi P., Cao Y., Shi B., Shen H., Zhao S., Gao Y., Chi H., Wang L., Shi Y. (2025). Isolation and Purification of Novel Antioxidant Peptides from Mussel (*Mytilus edulis*) Prepared by Marine Bacillus velezensis Z-1 Protease. Mar. Drugs.

[13] Kardani K., Bolhassani A. (2021). Antimicrobial/anticancer peptides: Bioactive molecules and therapeutic agents. Immunotherapy.

[14] Jin S.K., Choi J.S., Yim D.G. (2020). Hydrolysis Conditions of Porcine Blood Proteins and Antimicrobial Effects of Their Hydrolysates. Food Sci. Anim. Resour..

[15] Tkaczewska J., Jamróz E., Piątkowska E., Borczak B., Kapusta-Duch J., Morawska M. (2019). Furcellaran-coated microcapsules as carriers of Cyprinus carpio skin-derived antioxidant hydrolysate: An in vitro and in vivo study. Nutrients.

[16] Li Y., Li J., Lin S.-J., Yang Z.-S., Jin H.-X. (2019). Preparation of antioxidant peptide by microwave-assisted hydrolysis of collagen and its protective effect against H_2_O_2_-induced damage of RAW264. 7 cells. Mar. Drugs.

[17] Piao M., Tu Y., Zhang N., Diao Q., Bi Y. (2023). Advances in the Application of Phytogenic Extracts as Antioxidants and Their Potential Mechanisms in Ruminants. Antioxidants.

[18] Barnabé M., Vicente L., Martins K.V.C., Lacerda G.F., Rodrigues E., Oliveira L.A., Dias K.A., Pereira S.M.S., José V., Dias M. (2025). Soybean Flour Fortified with *Gryllus assimilis* Powder to Increase Iron Bioavailability Improves Gut Health and Oxidative Balance In Vivo. Nutrients.

[19] Box A., Capó X., Tejada S., Catanese G., Grau A., Deudero S., Sureda A., Valencia J.M. (2020). Reduced Antioxidant Response of the Fan Mussel *Pinna nobilis* Related to the Presence of *Haplosporidium pinnae*. Pathogens.

[20] de Freitas A.C., Reolon H.G., Abduch N.G., Baldi F., Silva R.M.O., Lourenco D., Fragomeni B.O., Paz C.C.P., Stafuzza N.B. (2024). Proteomic identification of potential biomarkers for heat tolerance in Caracu beef cattle using high and low thermotolerant groups. BMC Genom..

[21] Dong H., Liu M., Wang L., Liu Y., Lu X., Stagos D., Lin X., Liu M. (2021). Bromophenol Bis (2,3,6-Tribromo-4,5-dihydroxybenzyl) Ether Protects HaCaT Skin Cells from Oxidative Damage via Nrf2-Mediated Pathways. Antioxidants.

[22] Bah C.S.F., Bekhit A.E.D.A., Carne A., McConnell M.A. (2015). Composition and biological activities of slaughterhouse blood from red deer, sheep, pig and cattle. J. Sci. Food Agric..

[23] Xin X.Y., Ruan C.H., Liu Y.H., Jin H.N., Park S.K., Hur S.J., Li X.Z., Choi S.H. (2024). Identification of novel antioxidant and anti-inflammatory peptides from bovine hemoglobin by computer simulation of enzymolysis, molecular docking and molecular dynamics. Curr. Res. Food Sci..

[24] Sanchez-Reinoso Z., Todeschini S., Thibodeau J., Ben Said L., Fliss I., Bazinet L., Mikhaylin S. (2022). Impact of Pulsed Electric Fields and pH on Enzyme Inactivation and Bioactivities of Peptic Hydrolysates Produced from Bovine and Porcine Hemoglobin. Foods.

[25] Sun Q., Shen H., Luo Y. (2011). Antioxidant activity of hydrolysates and peptide fractions derived from porcine hemoglobin. J. Food Sci. Technol..

[26] Lu T., Piao X., Zhang Q., Wang D., Piao X., Kim S. (2010). Protective effects of Forsythia suspensa extract against oxidative stress induced by diquat in rats. Food Chem. Toxicol..

[27] Hu B., Chen Y., Hu H., Li C., Yang Y., He L., Su Z. (2014). The Antioxidant Activity of Porcine Blood Hydrolysate. J. Nucl. Agric. Sci..

[28] Higuchi M., Kobayashi S., Kawasaki N., Hamaoka K., Watabiki S., Orino K., Watanabe K. (2007). Protective effects of wheat bran against diquat toxicity in male Fischer-344 rats. Biosci. Biotechnol. Biochem..

[29] Livak K.J., Schmittgen T.D. (2001). Analysis of relative gene expression data using real-time quantitative PCR and the 2(-Delta Delta C(T)) Method. Methods.

[30] Ilie D., Iosageanu A., Craciunescu O., Seciu-Grama A.M., Sanda C., Oancea F. (2022). Free Radical Scavenging, Redox Balance and Wound Healing Activity of Bioactive Peptides Derived from Proteinase K-Assisted Hydrolysis of *Hypophthalmichthys molitrix* Skin Collagen. Food Technol. Biotechnol..

[31] Obianwuna U.E., Oleforuh-Okoleh V.U., Wang J., Zhang H.J., Qi G.H., Qiu K., Wu S.G. (2022). Natural Products of Plants and Animal Origin Improve Albumen Quality of Chicken Eggs. Front. Nutr..

[32] Xiang Z., Xue Q., Gao P., Yu H., Wu M., Zhao Z., Li Y., Wang S., Zhang J., Dai L. (2023). Antioxidant peptides from edible aquatic animals: Preparation method, mechanism of action, and structure-activity relationships. Food Chem..

[33] Hao Y., Xing L., Wang Z., Cai J., Toldrá F., Zhang W. (2023). Study on the anti-inflammatory activity of the porcine bone collagen peptides prepared by ultrasound-assisted enzymatic hydrolysis. Ultrason Sonochem..

[34] Nikhita R., Sachindra N.M. (2021). Optimization of chemical and enzymatic hydrolysis for production of chicken blood protein hydrolysate rich in angiotensin-I converting enzyme inhibitory and antioxidant activity. Poult. Sci..

[35] Outman A., Deracinois B., Flahaut C., Diab M.A., Dhaouefi J., Gressier B., Eto B., Nedjar N. (2023). Comparison of the Bioactive Properties of Human and Bovine Hemoglobin Hydrolysates Obtained by Enzymatic Hydrolysis: Antimicrobial and Antioxidant Potential of the Active Peptide α137-141. Int. J. Mol. Sci..

[36] Verma A.K., Chatli M.K., Kumar P., Mehta N. (2022). Assessment of quality attributes of porcine blood and liver hydrolysates incorporated pork loaves stored under aerobic and modified atmospheric packaging. J. Food Sci. Technol..

[37] Verma A.K., Chatli M.K., Kumar P., Mehta N. (2018). Effects of inclusion of porcine blood hydrolysate on physico-chemical quality, oxidative and microbial stability of pork batter stored at (4  ±  1 °C). J. Food Sci. Technol..

[38] Xue D., Jiang S., Zhang M., Shan K., Lametsch R., Li C. (2024). The efficiency and safety evaluation of hemoglobin hydrolysate as a non-heme iron fortifier. Food Sci. Hum. Wellness.

[39] Bechaux J., Gatellier P., Le Page J.-F., Drillet Y., Sante-Lhoutellier V. (2019). A comprehensive review of bioactive peptides obtained from animal byproducts and their applications. Food Funct..

[40] Besharati M., Lackner M. (2023). Bioactive peptides: A review. EuroBiotech J..

[41] Wu H.-C., Pan B.S., Chang C.-L., Shiau C.-Y. (2005). Low-molecular-weight peptides as related to antioxidant properties of chicken essence. J. Food Drug Anal..

[42] Serena-Romero G., Ignot-Gutiérrez A., Conde-Rivas O., Lima-Silva M.Y., Martínez A.J., Guajardo-Flores D., Cruz-Huerta E. (2023). Impact of In Vitro Digestion on the Digestibility, Amino Acid Release, and Antioxidant Activity of Amaranth (*Amaranthus cruentus* L.) and Cañihua (Chenopodium pallidicaule Aellen) Proteins in Caco-2 and HepG2 Cells. Antioxidants.

[43] Zhu Y., Lao F., Pan X., Wu J. (2022). Food Protein-Derived Antioxidant Peptides: Molecular Mechanism, Stability and Bioavailability. Biomolecules.

[44] Kleekayai T., O’Neill A., Clarke S., Holmes N., O’Sullivan B., FitzGerald R.J. (2022). Contribution of Hydrolysis and Drying Conditions to Whey Protein Hydrolysate Characteristics and In Vitro Antioxidative Properties. Antioxidants.

[45] Boulebd H., Spiegel M. (2023). Computational assessment of the primary and secondary antioxidant potential of alkylresorcinols in physiological media. RSC Adv..

[46] Xu B., Dong Q., Yu C., Chen H., Zhao Y., Zhang B., Yu P., Chen M. (2024). Advances in Research on the Activity Evaluation, Mechanism and Structure-Activity Relationships of Natural Antioxidant Peptides. Antioxidants.

[47] Sampath Kumar N.S., Nazeer R.A. (2012). In vivo antioxidant activity of peptide purified from viscera protein hydrolysate of horse mackerel (*Magalaspis cordyla*). Int. J. Food Sci. Technol..

[48] Han K.H., Shimada K., Hayakawa T., Yoon T.J., Fukushima M. (2014). Porcine Splenic Hydrolysate has Antioxidant Activity in vivo and in vitro. Korean J. Food Sci. Anim. Resour..

[49] Wu H., Xi H., Lai F., Ma J., Liu H. (2018). Chemical and cellular antioxidant activity of flavone extracts of *Labisia pumila* before and after in vitro gastrointestinal digestion. RSC Adv..

[50] Fitian A.I., Nelson D.R., Liu C., Xu Y., Ararat M., Cabrera R. (2014). Integrated metabolomic profiling of hepatocellular carcinoma in hepatitis C cirrhosis through GC/MS and UPLC/MS-MS. Liver Int..

[51] Sun J., Zhou C., Cao J., He J., Sun Y., Dang Y., Pan D., Xia Q. (2022). Purification and Characterization of Novel Antioxidative Peptides From Duck Liver Protein Hydrolysate as Well as Their Cytoprotection Against Oxidative Stress in HepG2 Cells. Front. Nutr..

[52] Chakrabarti S., Guha S., Majumder K. (2018). Food-Derived Bioactive Peptides in Human Health: Challenges and Opportunities. Nutrients.

[53] Qu J., Pei H., Li X.Z., Li Y., Chen J.M., Zhang M., Lu Z.Q. (2024). Erythrocyte membrane biomimetic EGCG nanoparticles attenuate renal injury induced by diquat through the NF-κB/NLRP3 inflammasome pathway. Front. Pharmacol..

[54] He C., Cai G., Jia Y., Jiang R., Wei X., Tao N. (2025). Effect of Diquat on gut health: Molecular mechanisms, toxic effects, and protective strategies. Front. Pharmacol..

[55] Guerrero-Hue M., Rayego-Mateos S., Vázquez-Carballo C., Palomino-Antolín A., García-Caballero C., Opazo-Rios L., Morgado-Pascual J.L., Herencia C., Mas S., Ortiz A. (2020). Protective Role of Nrf2 in Renal Disease. Antioxidants.

[56] Batty M., Bennett M.R., Yu E. (2022). The Role of Oxidative Stress in Atherosclerosis. Cells.

[57] Yang B., Lin Y., Huang Y., Shen Y.Q., Chen Q. (2024). Thioredoxin (Trx): A redox target and modulator of cellular senescence and aging-related diseases. Redox Biol..

[58] Patinen T., Adinolfi S., Cortés C.C., Härkönen J., Jawahar Deen A., Levonen A.L. (2019). Regulation of stress signaling pathways by protein lipoxidation. Redox Biol..

