# Supplementary material for: Evaluation of the In Vivo Antioxidant Capacity of Sheep Hemoglobin Hydrolysate in a Rat Model
Source: Biology (Basel). 2026 Jul 23;15(15):1227. doi: 10.3390/biology15151227 (PMC13464934; doi:10.3390/biology15151227)
Supplement: Supplementary file 1 [file biology-15-01227-s001.zip › biology-4392018-supplementary.pdf]

Table S1. Effects of sheep hemoglobin hydrolysate on feed intake and body weight in diquat challenged rats (g)

Items[1]	DG[2]	VC	SHH	*P*-value
		FI		
1-5d	20.47±0.76	21.00±0.57	19.65±0.77	0.4467
6-10d	19.96±1.17	21.07±0.73	19.78±0.42	0.5313
11-15d	24.45±1.67	23.99±0.71	22.00±0.37	0.2983
16-21d	24.40±1.27	22.95±0.26	21.66±1.00	0.2034
		BW		
1d	256.72±2.57	253.81±6.75	255.36±9.57	0.9625
10d	259.03±3.11	253.82±6.63	255.87±8.98	0.8773
21d	260.49±5.25	254.16±7.52	256.50±9.13	0.8513

[1] Data are presented as mean ± standard error. Values in the same column with different lowercase superscript letters (a, b, and c) are significantly different ($p < 0.05$), whereas those with no letter display are not significantly different ($p > 0.05$), n=6. [2] Abbreviations: DG, diquat-challenged group; VC, standard control group; SHH, sheep hemoglobin hydrolysate group; FI, feed intake; BW, body weight.

Table S2. Effects of sheep hemoglobin hydrolysate on organs weight and index in diquat challenged rats

Items[1]	DG[2]	VC	SHH	*P*-value
Liver weight (g)	6.99±0.55	6.51±0.08	6.33±0.24	0.4006
Liver index (%)	2.95±0.09	2.98±0.08	2.87±0.07	0.5989
Kidney weight (g)	1.70±0.11	1.57±0.03	1.60±0.05	0.3994
Renal index (%)	0.73±0.02	0.71±0.02	0.73±0.02	0.8872
Spleen weight (g)	0.40±0.02	0.39±0.02	0.41±0.03	0.8937
Spleen index (%)	0.17±0.01	0.18±0.01	0.18±0.02	0.7931
Ileum weight (g)	1.76±0.17	2.07±0.15	2.22±0.17	0.4332
Ileum index (%)	0.80±0.06	0.95±0.08	1.00±0.06	0.1495

[1] Data are presented as mean ± standard error. Values in the same column with different lowercase superscript letters (a, b, and c) are significantly different ($p < 0.05$), whereas those with no letter display are not significantly different ($p > 0.05$), n=6.[2] Abbreviations: DG, diquat-challenged group; VC, standard control group; SHH, sheep hemoglobin hydrolysate group
